# Effects of fungal-assisted algal harvesting through biopellet formation on pesticides in water

**DOI:** 10.1007/s10532-018-9852-y

**Published:** 2018-09-01

**Authors:** Malin Hultberg, Hristina Bodin

**Affiliations:** 1 Department of Biosystems and Technology, Swedish University of Agricultural Sciences, P.O. Box 103, 230 53 Alnarp, Sweden; 2 Division of Natural Sciences, Kristianstad University, Kristianstad, Sweden

**Keywords:** *Aspergillus niger*, Bioremediation, *Chlorella vulgaris*, Emerging pollutants, Water quality

## Abstract

Recent research has demonstrated the potential of using filamentous fungi to form pellets with microalgae (biopellets), in order to facilitate harvesting of microalgae from water following algae-based treatment of wastewater. In parallel, there is a need to develop techniques for removing organic pollutants such as pesticides and pharmaceuticals from wastewater. In experiments using the microalga *Chlorella vulgaris*, the filamentous fungus *Aspergillus niger* and biopellets composed of these microorganisms, this study investigated whether fungal-assisted algal harvesting can also remove pesticides from contaminated water. A mixture of 38 pesticides was tested and the concentrations of 17 of these were found to be reduced significantly in the biopellet treatment, compared with the control. After harvesting, the concentration of total pesticides in the algal treatment did not differ significantly from that in the control. However, in the fungal treatment and biopellet treatment, the concentration was significantly lower (59.6 ± 2.0 µg/L and 56.1 ± 2.8 µg/L, respectively) than in the control (66.6 ± 1.0 µg/L). Thus fungal-assisted algal harvesting through biopellet formation can also provide scope for removing organic pollutants from wastewater, with removal mainly being performed by the fungus.

**Electronic supplementary material:**

The online version of this article (10.1007/s10532-018-9852-y) contains supplementary material, which is available to authorized users.

## Introduction

The world’s population is increasing rapidly and waste streams such as municipal wastewater are being produced in ever increasing quantities. This has generated interest in developing sustainable technologies with low energy demand for municipal wastewater treatment that allow recirculation of resources, such as nutrients needed for crop production. One possibility is algae-based technologies such as high-rate algal ponds, which are well-known for removal of inorganic nutrients from wastewater (Oswald 1988; Shilton et al. 2012). Another positive aspect of using microalgae for wastewater treatment is that the biomass obtained after treatment can be an economic benefit if used within the emerging biofuel sector (Gentili 2014).

A current bottleneck in using microalgae for treatment of wastewater is the harvesting step, which requires methods such as filtration, chemical flocculation and centrifugation. These harvesting methods may account for as much as 20–30% of the total algal biomass production costs and are also very energy-demanding (Uduman et al. 2010). Recent research has indicated the potential for using filamentous fungi to form pellets with microalgae (biopellets) in order to advance the sustainability and economic feasibility of producing and harvesting microalgal biomass in wastewater (Zhang and Hu 2012; Bhattacharya et al. 2017).

In parallel, studies over the past decade have shown that conventional wastewater treatment processes only partly remove organic pollutants such as pesticides and pharmaceuticals (Jones et al. 2005; Hollender et al. 2009; Pereira et al. 2015). These substances are now frequently detected in aquatic ecosystems (Loos et al. 2009; Masiá et al. 2013), and in groundwater and drinking water (Loos et al. 2010a). Thus, there is also a need to develop methods for removing organic pollutants from water, especially methods with high efficiency when treating dilute effluents. European Union Directive 2013/39/EU set by the European Commission (EC 2013) highlights this need in order to combat priority compounds, which include several pesticides. Methods such as ozonation and active carbon filtration have been demonstrated to be efficient in removing organic pollutants (Hollender et al. 2009). In some situations, wastewater treatment techniques based on biological processes could also be a low-cost alternative.

One sustainable and low-cost method for treatment of water contaminated with organic pollutants, such as pesticides, is through use of naturally occurring microorganisms. Reductions in organic pollutant concentrations in water can be achieved through initial and rapid biosorption onto the microorganism biomass and through biodegradation of the pollutant (Fomina and Gadd 2014). Microalgae and fungi have both been demonstrated to effectively remove pesticides from water (Cai et al. 2007; Pinto et al. 2012; Yakout 2014; Hultberg et al. 2016). For microalgae, the effect can be partly explained by a synergistic relationship between the photosynthetic microalgae and heterotrophic bacteria degrading the pollutant (Muñoz and Guieysse 2006). For fungi, certain species are of high interest for bioremediation of media contaminated with organic pollutants due to their production of extracellular enzymes, such as laccases, capable of degrading recalcitrant xenobiotics (Viswanath et al. 2014). Removal of pesticides from water by microorganisms generally follows first-order kinetics, where the removal rate is directly correlated to the pollutant concentration (Cai et al. 2007). The pesticide levels normally found in contaminated aquatic ecosystems are low for individual substances, often at levels below 5 µg/L (Kreuger et al. 2010), but still well above the maximum acceptable limits stated in the EU watchlist of priority substances (EC 2015).

The use of filamentous fungi for harvesting microalgae, through formation of biopellets, is currently being intensely researched (Bhattacharya et al. 2017) and has high potential for being developed into a sustainable technology. The aim of the present study was to investigate the effect of this harvesting process on pesticide removal from contaminated water. The experiments were performed at low pesticide concentrations using a mixture of pesticides from different substance groups and the microalga *Chlorella vulgaris* and the filamentous fungus *Aspergillus niger*.

## Materials and methods

### Microorganisms

The microalgal species *Chlorella vulgaris* strain 211/11B from CCAP-SAMS (Culture Collection of Algae and Protozoa, The Scottish Association for Marine Science), Scotland, and the filamentous fungus *Aspergillus niger* ATCC^®^ 16888™ from the American Type Culture Collection were used in the study.

The microalgal strain was cultivated in BG-11 medium (Zhang and Hu 2012). The algal culture was started by inoculating BG-11 medium with 10% (v/v) of a *C. vulgaris* culture taken from a 4-day-old algal culture. The culture was maintained in a greenhouse at 20 °C (photoperiod 16 h) and illumination of 50 µmol/m^2^ s (PAR). The culture was aerated (0.3 vvm) to prevent the algal cells from settling. After 4 days, the number of algal cells was determined by counting in a Bürkner chamber and a stock solution containing 2.0 × 10^7^ cells/mL was made using BG-11 medium.

Fungal spores were cultivated on petri plates with potato dextrose agar (PDA) at room temperature for 10 days. The spores were harvested by applying 3 × 10 mL of glucose solution (20 g/L) directly onto the PDA plates using a sterile syringe. The spore solution was filtered through a nylon filter (mesh size 100 µm). The number of fungal spores was determined by counting in a Bürkner chamber and a stock solution containing 3.9 × 10^5^ fungal spores/mL was prepared.

Both the algal and the fungal stock solution were kept at 4 °C in darkness until the start of the experiment on the same day. Biopellets were formed by mixing equal amounts of microalgae stock solution and fungal stock solution (Zhang and Hu 2012).

### Selected pesticides

A pesticide mixture (Mix M2101/1B, Analytical Reference Material, Restek, USA) was obtained from the Centre for Chemical Pesticides (CKB), Swedish University of Agricultural Sciences, Ultuna. This mixture contained one plant growth regulator (trinexapac ethyl) and the following 37 pesticides: acetamiprid, carbofuran, carfentrazone ethyl, chlorfenvinphos, cyanazine, clomazone, cloridazon, cyazofamid, cyprodinil, difenoconazole, ethofumesate, fenpropidin, fludioxonil, flurprimidol, flurtamone, flusilazole, flutriafol, fuberidazole, hexazinone, imidachloprid, mandipropamid, metalaxyl, metamitron, metazachlor, metolachlor, metrafenone, penconazole, phenmedipham, pirimicarb, propamocarb, propyzamide, protioconazole-destio, pyroxsulam, quinmerac, spiroxamin, terbuthylazine and triticonazole. The initial concentration in the treatments and properties of these pesticides are described in Online Resource 1. The mixture was diluted in sterile distilled water and the solution was stirred for 5 min before being used in the experiments. The concentrations of the pesticides in the solution were determined by method OMK 57 (Jansson and Kreuger 2010), as described below. Total concentration of pesticides was then determined as sum of individual pesticides. Total pesticide concentration at the start of the experiments was 72.7 ± 1.8 µg/L in all treatments.

### Experimental design

Four treatments were included in the study: a sterile control treatment, a microalgal treatment, a fungal treatment and a treatment with biopellets (Table 1).

**Table 1:** Dry weight biomass, specific growth rate (SGR) and pH in the different treatments. Mean and standard deviation are shown

Treatment	Content	Biomass (dwt, mg/L)		PH		SGR (%/h)
		Initial	Final	Initial	Final	
Control	BG-11, glucose solution, pesticide solution	0	0	4.0 ± 0.0	4.0 ± 0.0a	0
Microalgal	Microalgal stock solution, glucose solution, pesticide solution	174.2 ± 3.9	353.6 ± 10.2a*	4.0 ± 0.0	4.2 ± 0.2a	1.05 ± 0.0a
Fungal	BG-11, fungal stock solution, pesticide solution	42.9 ± 9.0	540.0 ± 27.5b	4.0 ± 0.0	3.1 ± 0.1b	3.78 ± 0.3b
Biopellet	Microalgal and fungal stock solution, pesticide solution	181.6 ± 8.5	532.9 ± 21.8b	4.0 ± 0.0	3.0 ± 0.1b	1.59 ± 0.1c

*Values within columns followed by different letters are significantly different (P < 0.05, Tukey’s test)

The control treatment contained equal amounts of BG-11, glucose solution and pesticide solution. The microalgal treatment contained equal amounts of algal stock solution, glucose solution and pesticide solution. The fungal treatment consisted of BG-11, fungal stock solution made in glucose and pesticide solution. The biopellet treatment consisted of algal stock solution, fungal stock solution and pesticide solution. The initial pH was adjusted to 4.0 in all treatments, using HCl, as this pH has been identified as optimal for biopellet formation and algal harvest by the fungus *A. niger* (Zhang and Hu 2012).

The treatments were placed on a horizontal shaker (100 rpm) at room temperature without additional light. Based on the time it takes to form biopellets (Zhang and Hu 2012), a treatment time of 68 h was applied. After this time, the treatments were removed from the shaker and the biomass produced was collected by filtration through GF/F filters (VWR International, filter no. 698). The control treatment was filtered in the same manner as the other treatments and the water samples for analysis of pesticide levels were stored at − 18 °C. Samples for analysis of chemical oxygen demand (COD) reduction and laccase activity were taken after 0, 24, 48 and 68 h.

### Analysis

#### Growth parameters

At the start and end of the experiment, dry weight biomass in all treatments was estimated by filtration. A similar volume of each sample was filtered through pre-dried and pre-weighed GF/B filters. The filters were dried at 65 °C for 2 h and re-weighed on a precision balance. The control treatment was filtered in the same manner as the other treatments. Specific growth rate (SGR) of the microorganisms used was calculated as (Lawton et al. 2013):

$${\text{SGR }} = \left[ {\ln (B_{f} /B_{i} ) \cdot t} \right]\, \cdot 100$$

where *B*_*f*_ and *B*_*i*_ are the final and initial dry weight biomass concentration (mg/L) and *t* is the number of hours in the batch experiment.

For determination of chemical oxygen demand (COD) in the treatments, samples were taken at the start of the experiment and after 24 h, 48 h and 68 h (the end of the experiment). Aliquots were removed from each replicate, filtered (GF/B filters) and concentration of COD was determined using Hach Lange LCK 014 (ISO 1989). Laccase activity was determined colorimetrically by detecting the product of oxidation of 2,6-dimethoxyphenol (DMP). The analysis was performed as described by Parenti et al. (2013) and the reaction mixture contained 0.45 mL of the filtered water samples and 0.5 mL of 10 mM DMP in 100 mM acetate buffer (pH 5). After 1 min of incubation at room temperature, the absorbance at 468 nm was measured using a DR1900 Hach Portable Spectrophotometer.

#### Pesticide analysis

The filtered and frozen water samples were sent to an accredited laboratory at CKB that is responsible for the Swedish national environmental monitoring programme for pesticides. The analytical method used, OMK 57, is based on online SPE extraction and liquid chromatography-tandem mass spectrometry (LC–MS/MS). This method permits detection of numerous pesticides at low concentrations, as described in detail by Jansson and Kreuger (2010), with recoveries higher than 70% and limit of quantification (LOQ) values at or below 10 ng/L for individual pesticides.

Absolute pesticide reduction due to treatment was estimated as the difference between initial concentration in the control treatment and final concentration in the different treatments. The relative pesticide reduction was calculated as:

$${\text{Relative reduction }} = \left( {\frac{{\left( {C_{initial} - C_{final} } \right)}}{{C_{initial} }}} \right) \times 100$$

where *C*_*initial*_ = mean initial concentration of a particular pesticide (µg/L) and *C*_*final*_ = final concentration of that pesticide in a specific treatment (µg/L).

#### Statistical analysis

The experiment was set up with three replicates in each treatment and the data obtained were analysed statistically using Minitab 16 for Windows. In order to inspect the data for homogeneity of variance and normal distribution, the Levene and Kolmogorov–Smirnov test, respectively, was used. One-way Anova followed by Tukey’s multiple comparison test was employed to test for effects of treatment on final concentrations of pesticides in the water. The significance level was set to *p *< 0.05 in all cases.

## Results and discussion

### Growth parameters

This study was performed under conditions previously identified as optimal for biopellet formation and algal harvest by the fungus *A. niger*, i.e. low pH, addition of a suitable carbon source and no additional light (Zhang and Hu 2012). From a practical perspective, these conditions should be applied after microalgal growth in wastewater, since the environment created is less suited for microalgal growth. However, biomass production was observed in all treatments except the control, which was kept under sterile conditions (Table 1). The observed biomass production in the microalgal treatment (Table 1) was probably because the microalgae in the stock solution were in their exponential growth phase when the experiment started. Specific growth rate was significantly different between the treatments, with the highest values observed for the fungal treatment and the lowest for the microalgal treatment (Table 1). This difference reflects the environmental conditions, which were suboptimal for the microalgae, whereas *A. niger* is well-known for growth at low pH and with capability for growth down to pH 2 (Pitt 1981).

**Fig. 1:**
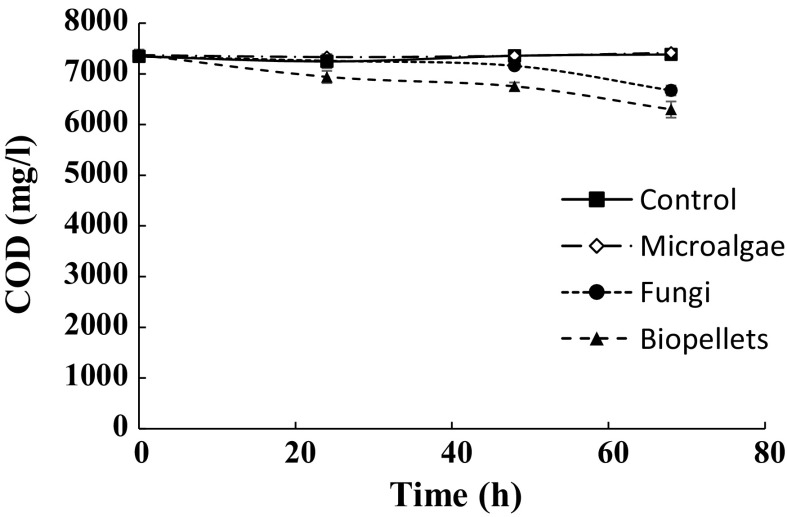
Decrease in chemical oxygen demand (COD) concentration over time in the treatments. Mean and standard deviation (bars) are shown

Fungal growth in treatments involving the fungus was also reflected in decreased COD concentration in those treatments (Fig. 1). After 68 h, the COD concentration had been lowered by 9.1% in the fungal treatment and 14.6% in the biopellet treatment, compared with the initial value. This decrease was significantly higher for the biopellet treatment than the fungal treatment, despite the final amount of biomass not differing between these treatments (Table 1). As *C. vulgaris* has the capability for mixotrophy (Lia et al. 2014), a possible explanation for the significantly higher decrease in COD concentration in the biopellet treatment is that the microalgal cells were protected from the unfavourable low pH environment when entrapped in the fungal biomass and showed heterotrophy in the absence of light. As expected, there was no decrease in COD concentration in the control treatment and the microalgal treatment.

A change in the initial pH was observed during the experiments, with a significant decrease in the fungal and biopellet treatments compared with the control (Table 1). *Aspergillus niger* is well-known for production of extracellular enzymes and citric acid, and is used commercially for production of the latter (Yakout 2014). The significantly lower pH values found in the fungal and biopellet treatments confirm that the fungus was metabolically active.

*Aspergillus niger* has also been suggested as a good source of laccase, a multicopper oxidase with several biotechnological applications (Tamayo Ramos et al. 2011). Fungal laccases are used for lignin degradation/modification during growth, but these enzymes have low substrate specificity and act upon a wide range of complex molecules (Rhodes 2014). They are therefore of interest for degradation of recalcitrant xenobiotics, and high production has been demonstrated in environments with low pH (Viswanath et al. 2014). In the present study, no production of extracellular laccases was observed in either of the treatments with the fungus when assayed with the substrate DMP. Different substrate specificity for the laccases produced by *A. niger* was observed in the study by Tamayo Ramos et al. (2011) and therefore production of laccase cannot be completely ruled out.

### Effects of treatments on total amount of pesticides in water

As mentioned, the total amount of pesticides in the water samples was 72.7 ± 1.8 µg/L when the experiment started. Removal over time due to abiotic factors was observed and, when the experiment ended after 68 h, the concentration of pesticides in the untreated sterile control was 66.6 ± 1.0 µg/L. At the end of the experiment, the total concentration of pesticides in the algal treatment was 67.3 ± 1.2 µg/L and did not differ significantly from that in the sterile control. In the fungal treatment and the biopellet treatment, the final total pesticide concentration was 59.6 ± 2.0 µg/L and 56.1 ± 2.8 µg/L, respectively, which was significantly lower than in the algal treatment and the sterile control. These results clearly indicate that, under conditions favourable for fungal-assisted algal harvesting, the fungal species was solely responsible for the pesticide reduction. It should also be pointed out that initial dry weight biomass of the microorganisms used for treatment in the present study (Table 1) was between 10- and 100-fold lower than the biomass amount used in activated sludge processes at wastewater treatment plants, which is normally within the range 2–13 g/L (Serrano et al. 2011; Falås et al. 2013). Thus, despite the low amounts of fungus and biopellets used in treatments, significant pesticide removal was observed.

In a previous study using a similar experimental set-up and focusing on the effect of microalgae, a significant decrease in pesticide concentrations in water was observed after 4 days of microalgal cultivation (Hultberg et al. 2016). That study was performed in an environment favourable for microalgal growth, with added light and neutral pH. As previously pointed out, one of the most interesting aspects of biopellet formation is its potential to assist in microalgal harvesting. Thus, the organic pollutant removal potential of fungal treatment observed in this study and that of actively growing microalgae reported in our previous study should be considered in combination. In parallel with the benefit of biomass production, microalgal-based wastewater treatment offers an interesting technique for removal of organic pollutants (Matamoros et al. 2015; Hultberg et al. 2016). The present study demonstrates that fungal-assisted microalgal harvesting based on biopellets has the potential to remove organic pollutants from wastewaters.

### Effect of treatments on individual pesticides

**Fig. 2:**
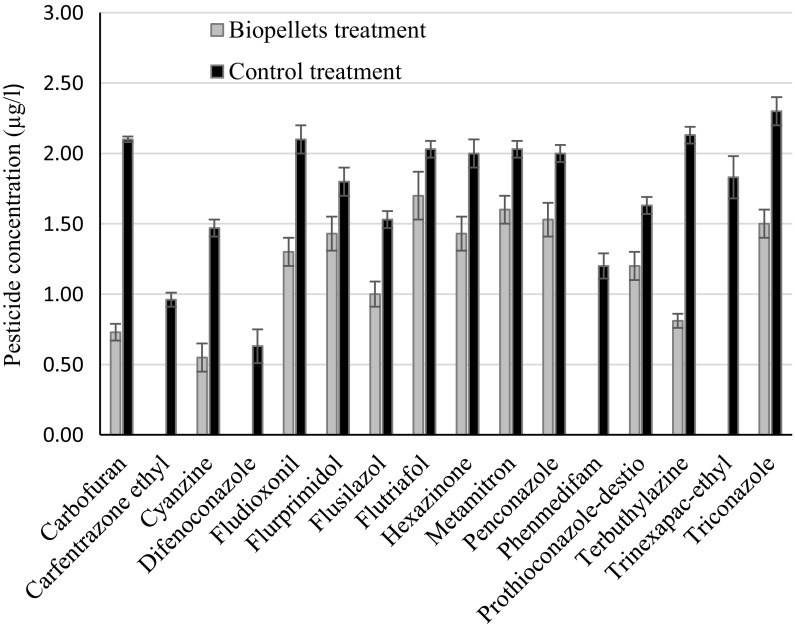
Concentrations of pesticides which were significantly decreased by the biopellet treatment compared with the concentration in the control treatment. Mean and standard deviation (bars) are shown

Of the 38 pesticides analysed, the concentrations of 17 were found to be reduced significantly after 68 h of biological treatment compared with a sterile control (Table 2). All of these were reduced in the biopellet treatment with the exception of cyazofamid, which was only reduced in the fungal treatment. Concentrations of the pesticides that were significantly removed in the biopellet treatment are shown in Fig. 2. All of the pesticides removed were associated with the biopellet/fungal treatments and none was only removed in the microalgal treatment.

**Table 2:** All pesticides with significantly lower concentrations compared with the control after treatment. Pesticides in italics had a removal rate of 50% or more compared with the control. Water containing 38 different pesticides was used in the study

Treatment	Biopellet	Fungal	Microalgal
	*Carbofuran* *Carfentrazone ethyl* *Cyanzine* * *Difenoconazole* Fludioxonil Flurprimidol Flusilazol Flutriafol Hexazinone Metamitron Penconazole *Phenmedifam* Prothioconazole-destio *Terbutylazine* *Trinexapac*-*ethyl* Triconazole	Carbofuran *Carfentrazone ethyl* *Cyanzine* Cyazofamid *Difenoconazole* Fludioxonil Flurprimidol * * Hexazinone * * *Phenmedifam* Prothioconazole-destio * Trinexapac-*ethyl* Triconazole	* *Carfentrazone ethyl* * * * Fludioxonil * * * * * * *Phenmedifam* * * * *
Total	16 (of 38)	12 (of 38)	3 (of 38)

*Not significantly lower compared with the control treatment (P < 0.05, Tukey’s test)

The pesticides difenoconazole, carfentrazone ethyl, phenmedipham and trinexapac ethyl were removed to below the detection limit after treatment with biopellets (Fig. 2). A previous study has shown complete, or very high, removal of these pesticides by biosorption to dead algal biomass (carfentrazone ethyl, phenmedipham) or by an actively growing microalgal culture (carfentrazone ethyl, difenconazole, trinexapac ethyl) (Hultberg et al. 2016). The results obtained in this study confirm that these pesticides are sensitive to biological treatment. The majority of pesticides that were significantly removed by the biopellet treatment belonged to the substance groups triazinones and triazoles, which are reported to be both toxic to aquatic organisms and frequently detected in aquatic environments (Loos et al. 2010a, b).

In the microalgal treatment, two of the significantly reduced pesticides, carfentrazone ethyl and phenmedipham, underwent relative removal of 50% or more compared with the control (Table 2). High removal of these two pesticides has previously been shown following short-term (1 h) exposure to dead microalgal biomass (Hultberg et al. 2016), suggesting rapid sorption to algal cell walls. Despite significant removal of these two pesticides, no effect was seen on total concentration of pesticides in the algal treatment and it should be pointed out that microalgal removal of pesticides was negligible under the conditions applied.

**Fig. 3:**
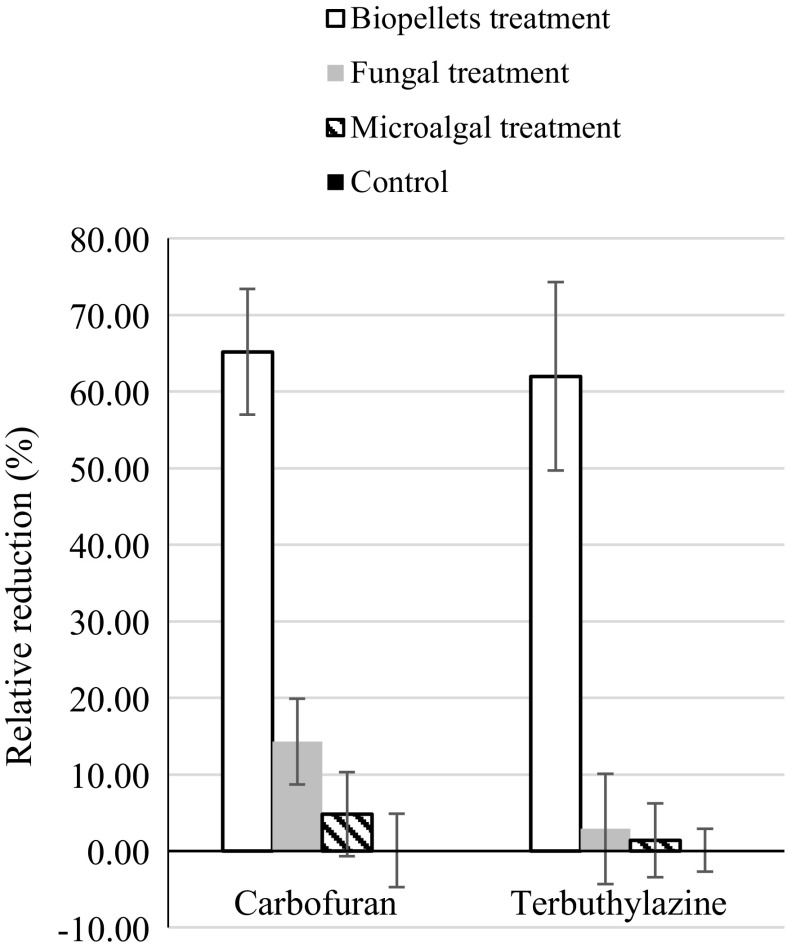
Relative reduction in carbofuran and terbuthylazine concentrations in the treatments. Mean and standard deviation (bars) are shown

The fungal treatment removed three additional pesticides (cyanazine, difenoconazole and trinexapac ethyl) by 50% or more. The biopellet treatment achieved a 50% decrease in the concentrations of two additional pesticides (carbofuran and terbuthylazine). Synergistic effects were indicated for these two pesticides, where relative removal of more than 50% was achieved by the biopellet treatment, while considerably lower removal was observed after the microalgal and fungal treatments (Fig. 3; Table 2). This indicates that the microalgal component in the biopellets may have had an effect in removal of specific pesticides. Within the context of persistence in surface water environments, terbuthylazine, which had a relative removal rate of 66% in the biopellet treatment, is one of the most frequently detected pesticides (Loos et al. 2010a, b).

## Conclusions

Algae-based technologies for treatment of wastewater are of interest, but harvesting of the microalgae is a bottleneck. A promising aspect of biopellet formation is its potential to assist in microalgal harvesting. A significant decrease in pesticide concentrations in water was observed after treatment with the filamentous fungus *Aspergillus niger* or with biopellets composed of the microalga *Chlorella vulgaris* and *A. niger*. Based on these findings, it can be concluded that fungal-assisted algal harvesting through biopellet formation increases the scope for removal of organic pollutants from wastewater. Under the conditions applied here, the fungal species was solely responsible for the pesticide reduction.

## Electronic supplementary material

Below is the link to the electronic supplementary material.

Supplementary material 1 (DOCX 30 kb)
