# Supplementary material for: Effects of fungal-assisted algal harvesting through biopellet formation on pesticides in water
Source: Biodegradation. 2018 Sep 1;29(6):557–65. doi: 10.1007/s10532-018-9852-y (PMC6245101; doi:10.1007/s10532-018-9852-y)
Supplement: Supplementary file 1 — Supplementary material 1 (DOCX 30 kb) [file 10532_2018_9852_MOESM1_ESM.docx]

**Supplemental Table 1.** The initial concentration and physicochemical properties of the pesticides included in the present study. Physical property data related to water contamination potential are compiled from PAN Pesticide Database (<http://www.pesticideinfo.org/Search_Chemicals.jsp>).

| **Pesticide** | **Substance group** | **Molecular mass** | **Initial conc.**  **(µg/L)** | **Water solubility**  **(mg/L)** | **Koc** | **Hydrolysis Half-live (Days)** | **Aerobic Soil Half-life (Days)** | **Anaerobic Soil Half-life (Days)** |
| --- | --- | --- | --- | --- | --- | --- | --- | --- |
| **Acetamiprid** | Neonicotinoid  (I*) | 222.7 | 2.3±0.1 | 3660.0 | 343.0 | 35.0 | 10.0 | 330.0 |
| **Carbofuran** | Carbamate  (I) | 221.3 | 0.6±0.1 | 351.0 | 25.0 | 18.0 | 22.0 | 20.0 |
| **Carfentrazone ethyl** | Triaolinone  (H) | 412.2 | 1.0±0.0 | 22.0 | 866.0 | 13.7 | 0.5 | -** |
| **Chlorfenvinphos** | Organophosphate  (I) | 359.6 | 1.9±0.0 | 145.0 | 680.0 | 125.0 | 40.0 | - |
| **Chloridazone** | Pyridazinone  (H) | 221.6 | 2.5±0.2 | 380.0 | 13800 | 30.0 | 124.0 | 489.0 |
| **Clomazone** | Isoxanzolidinone  (H) | 239.7 | 1.9±0.1 | 1100.0 | 244.0 | 34.0 | 66.0 | 19.0 |
| **Cyanazine** | Triazine  (H) | 240.7 | 1.6±0.1 | 155.0 | 188.0 | 3680.0 | 15.0 | 108.0 |
| **Cyazofamid** | Cyanoimidazole  (F) | 324.8 | 1.7±0.0 | 0.1 | 1780.0 | 25.0 | 10.0 | - |
| **Cyprodinil** | Anilinopyrimidine (F) | 225.3 | 0.5±0.0 | 16.0 | 1470.0 | 32.0 | 126.0 | 183.0 |
| **Difenoconazole** | Triazole  (F) | 406.3 | 0.5±0.0 | 15.0 | 6120.0 | 1730.0 | 318.0 | 361.0 |
| **Ethofumesate** | Benzofuran  (H) | 286.3 | 1.8±0.1 | 50.0 | 150.0 | 2900.0 | 93.0 | - |
| **Fenpropidin** | Unclassified  (F) | 273.5 | 1.5±0.0 | 530.0 | 3808.0 | - | 90.0 | - |
| **Fludioxonil** | Phenylpyrrole  (F) | 248.2 | 2.3±0.2 | 2.0 | 1610 | 30.0 | 102.0 | 365.0 |
| **Flurprimidol** | Pyrimidinyl carbinol (H) | 312.3 | 2.1±0.1 | 127.0 | 314.0 | 153000 | - | 3620 |
| **Flurtamone** | Pyridazinone  (H) | 333.3 | 2.4±0.1 | 10.7 | 329.0 | - | 56.0 | - |
| **Flusilazole** | Triazole  (F) | 315.4 | 1.4±0.1 | 41.9 | 1664 | - | 300.0 | - |
| **Flutriafol** | Triazole  (F) | 301.3 | 2.2±0.1 | - | 255.0 | 30.0 | 365.0 | 188.0 |
| **Fuberidazole** | Benzimidazole.  (F) | 184.2 | 2.5±0.1 | 71.0 | 605.0 | - | 6.0 | - |
| **Hexazinone** | Triazinone  (H) | 252.3 | 2.3±0.1 | 29800.0 | 642.0 | 56.0 | 222.0 | 232.0 |

| **Imidacloprid** | Neonicotinoid  (I) | 255.6 | 2.3±0.1 | 514.0 | 262.0 | 30.0 | 997.0 | 27.0 |
| --- | --- | --- | --- | --- | --- | --- | --- | --- |
| **Quinmerak** | Quinoline  (H) | 221.6 | 2.4±0.1 | 107000.0 | 86.0 | - | 30.0 | - |
| **Mandipropamid** | Mandelamide  (F) | 411.9 | 2.0±0.0 | 4.0 | 859.0 | 30.0 | 44.0 | 169.0 |
| **Metalaxyl** | Phenylamide  (F) | 279.3 | 2.3±0.1 | 8410.0 | 163.0 | 1000.0 | 62.0 | 68.0 |
| **Metamitron** | Triazinone  (H) | 202.2 | 2.4±0.1 | 1770.0 | 80.7 | 480.0 | 30.0 |  |
| **Metazachlor** | Chloroacetamide  (H) | 277.8 | 2.2±0.1 | 450.0 | 134.0 | - | 8.6 | - |
| **Metolachlor** | Chloroacetamide  (H) | 283.8 | 2.3±0.1 | 493.0 | 190.0 | 200.0 | 26.0 | 61.0 |
| **Metrafenon** | Benzophenone  (F) | 409.3 | 0.6±0.0 | 0.5 | 3105.0 | - | 250.6 | - |
| **Penconazole** | Triazole  (F) | 284.2 | 2.0±0.1 | 73.0 | 2205.0 | - | 197.0 | - |
| **Phenmedipham** | Bis-carbamate  (H) | 300.3 | 1.4±0.1 | 6.0 | - | 1.0 | 54.0 | 47.0 |
| **Pirimicarb** | Carbamate  (I) | 238.4 | 2.2±0.1 | 3100.0 | 388.0 | - | 86.0 | - |
| **Propamocarb** | Carbamate  (F) | 188.3 | 2.2±0.1 | 101000 | 619.0 | 30.0 | 77.0 | 92.0 |
| **Propyzamide** | Benzamide  (H) | 256.1 | 2.2±0.2 | 13.0 | 889.0 | 42.0 | 392.0 | 762.0 |
| **Protioconazole-destio** | Triazolinthione  (F) | 344.3 | 1.8±0.0 | - | - | - | - | - |
| **Pyroxsulam** | Triazolopyrimidine sulfonamide (H) | 434.4 | 1.5±0.1 | 3200.0 | 32.0 | 32.0 | 19.0 | 32.0 |
| **Quinmerac** | Quinoline  (H) | 221.6 | 2.4±0.1 | 107000.0 | 86.0 | - | 30.0 | - |
| **Spiroxamin** | Morpholine  (F) | 297.5 | 1.3±0.1 | 405.0 | 2415.0 | - | 25.0 | - |
| **Terbuthylazine** | Triazine  (H) | 229.7 | 2.4±0.1 | 6.6 | 219.0 | - | 76.7 | - |
| **Trinexapac ethyl** | Cyclohexanecarb  oxylate der. (Plant growth regulator) | 252.3 | 2.0±0.1 | 1400 | 440.0 | 456.0 | - | 13.0 |
| **Triticonazole** | Triazole  (F) | 317.8 | 2.4±0.1 | 8.0 | 523.0 | 30.0 | 220.0 | 235.0 |

* I Insecticide, F Fungicide, H Herbicide

** Data not found in the PAN Pesticide Database
